# ‘I don`t need an eye check-up’. A qualitative study using a behavioural model to understand treatment-seeking behaviour of patients with sight threatening diabetic retinopathy (STDR) in India

**DOI:** 10.1371/journal.pone.0270562

**Published:** 2023-06-15

**Authors:** Shuba Kumar, Rani Mohanraj, Rajiv Raman, Geetha Kumar, Sanjay Luvies, Shivani Sunil Machhi, Subhratanu Chakrabarty, Janani Surya, Radha Ramakrishnan, Dolores Conroy, Sobha Sivaprasad

**Affiliations:** 1 Social Science Department, Samarth, Chennai, Tamil Nadu, India; 2 Department of Ophthalmology, Sankara Nethralaya, Chennai, Tamil Nadu, India; 3 Department of Ophthalmology, Giridhar Eye Institute, Cochin, Kerala, India; 4 Department of Ophthalmology, Aditya Jyot Foundation for Twinkling Little Eyes, Mumbai, Maharashtra, India; 5 Department of Ophthalmology, VMA Netra Niramay Niketan, Haldia, West Bengal, India; 6 Department of Ophthalmology-NIHR Biomedical Research Centre, Moorfields Eye Hospital NHS Foundation Trust, London, United Kingdom; Province Health Service Directorate Surkeht, Technical Support Unit (TSU), NEPAL

## Abstract

Diabetic Retinopathy (DR) affects about 27% of patients with diabetes globally. According to the World Health Organization (WHO), DR is responsible for37 million cases of blindness worldwide. The SMART India study (October 2020-August 2021) documented the prevalence of diabetes, and DR in people40 years and above across ten Indian states and one Union Territory by conducting community screening. About 90% of people with sight threatening diabetic retinopathy (STDR) were referred from this screening study to eye hospitals for management, but failed to attend. This qualitative study, a component of the SMART India study, explored perceptions of referred patients regarding their susceptibility to eye related problems in diabetes and the benefits/barriers to seeking care. Perceived barriers from the viewpoint of ophthalmologists were also explored. Guided by the Health Beliefs Model (HBM), 20 semi structured interviews were carried out with consenting patients diagnosed with STDR. They included nine patients who had sought care recruited from eight eye hospitals across different states in India and eleven patients who did not seek care. Eleven ophthalmologists also participated. Four themes of analysis based on the HBM were, understanding of DR and its treatment, perceptions about susceptibility and severity, perceived barriers, perceived benefits and cues to action. Findings revealed poor understanding of the effects of diabetes on the eye contributing to low risk perception. Prohibitive costs of treatment, difficulties in accessing care services and poor social support were major barriers to seeking care. Ophthalmologists acknowledged that the absence of symptoms and the slow progressive nature of the disease deluded patients into thinking that they were fine. The study attests to the need for greater health literacy around diabetes, DR and STDR; for making treatment more affordable and accessible and for the development of effective patient education and communication strategies towards increasing compliance.

## Introduction

Diabetic retinopathy (DR) is a common microvascular complication of diabetes mellitus (DM) that can result in irreversible visual impairment if the sight threatening complications are not identified early and treated [1]. Clinically, DR can be graded as non-sight-threatening diabetic retinopathy (NSTDR) and sight-threatening diabetic retinopathy (STDR) [2, 3]. As STDR can be asymptomatic in the initial stages and timely treatment can prevent visual loss, retinal screening for DR is recommended for all people with diabetes [4]. Once DR is identified through the process of screening, the person is referred to an ophthalmologist equipped to provide treatment, if required. These treatments include retinal laser, intra-vitreal injections of drugs and/or vitreo-retinal surgery depending on the complexity of the retinopathy.

Globally, about 27% of individuals with diabetes have DR [5]. The prevalence of DR is estimated to be 31.6% in Africa [6]. According to the World Health Organization (WHO), DR is responsible for an estimated 37 million cases of blindness worldwide [7]. It is anticipated that the overall number of people with DR will rise due to the increasing prevalence of diabetes and the ageing and expanding global population.

DR-related blindness is on the decline in high-income countries (USA,UK) as a result of improved therapies, concerted public health efforts and increased public awareness about DR screening [8–10]. On the other hand, low and middle income countries (India, China) bear the brunt of the diabetes epidemic and its complications including DR. This is partly attributable to inadequate forward planning and limited access to high-quality affordable eye care [11]. Owing to its potential to cause vision loss and a commensurate decline in quality of life, its early detection and effective management will be the key to improving overall health outcomes in people with diabetes [12].

### DR in the Indian context

Despite the high prevalence of DM in India [13, 14] there exists wide variations in awareness about the disease in the general population. A report [15] suggested that approximately 50% of participants were not even aware of diabetes. Awareness about DR and other ocular related problems elicited through community based research studies carried out across different regions in India also revealed wide variations in awareness of DR ranging from 16.1% to 71.3% [16–19]. Of significance is the fact that among those diagnosed with DM, awareness about DR ranged from 17.01% to 93.2% [20–22]. Ramasamy et al. reported that although study participants were cognizant of DM, understanding of DR and its implications was poor [23]. The absence of symptoms; difficulties in doctor–patient interactions; burden of hospital visits and high costs were major deterrents to seeking care [24]. An earlier study of ours [25] that explored barriers to screening for DR, in addition to the above, also explored perceptions of health care providers wherein difficulties in communicating information about DR to less literate patients, heavy work pressure and silent progression of the disease were reported as major barriers to care delivery.

Another major challenge is the non-adherence to follow-up and treatment [26] and consequent adverse outcomes including irreversible visual loss [27]. Although this is partly explained by the lack of collaboration between screening and treatment services, there may be other reasons that could contribute to their non-attendance. All these findings highlight the need to improve strategies to enhance compliance.

### The SMART India study

The SMART India study (Multi centre statistical and economic modelling of risk based stratified and personalised screening of diabetes and its complication in India) was a cross-sectional screening study conducted between August 2018-June 2020 on people aged 40 years or above across 10 Indian states and 1 Union Territory (UT) [28]. Twenty tertiary level eye hospitals located in the above states and UT constituted the participating centres. The aims of the study were to assess the prevalence of diabetes and eye related complications, namely, DR and STDR towards developing risk models to identify and treat these conditions. Both urban and rural areas were included. The study found that out of the 42,416 people screened, a total of 7910 (18.8%)were identified to have diabetes. These included 5689 persons with known diabetes and 2221 with undiagnosed diabetes. The overall prevalence of DR was estimated at 12.5% and that of STDR was 4% [29].

People identified with DR in the study were referred to designated retinal departments in their respective sites where free treatment was offered. A total of 324 individuals were identified to have STDR but only 10% (n = 32) attended treatment despite repeated reminders given about the possible threat of losing their vision. To better understand this phenomenon of poor care seeking we carried out a qualitative study to explore the perceptions of patients with STDR regarding their understanding of and susceptibility to eye related problems and the benefits/barriers to seeking care. From the health care providers (HCPs) we explored what they believed were barriers to care seeking among STDR patients and also sought their insights into what could contribute to improved care seeking in such patients.

### Conceptual framework

**Fig 1:**
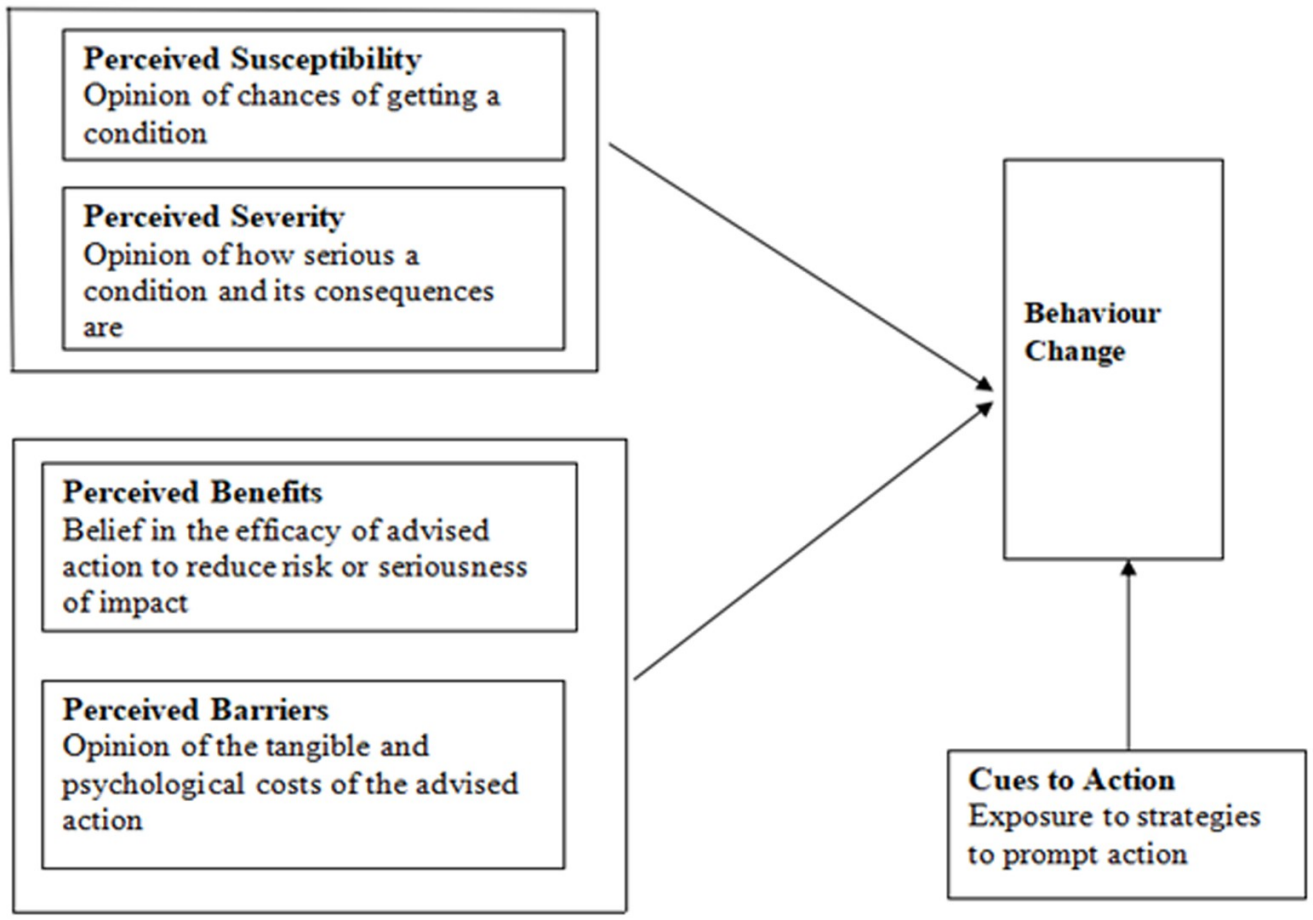
The Health Belief Model (HBM).

In undertaking this qualitative study we used the Health Belief Model (HBM) which is a social psychological health behaviour change) model developed to explain and predict health-related behaviours, particularly with regard to the use of health services [30, 31]. Employing a theoretical model such as the HBM we believed, was particularly well suited to our study because this model focuses on intra-personal factors, including risk-related beliefs which influence individuals’ health-related decision making [32]. Given that our study explored perceptions of disease risk, barriers to undergoing regular eye tests and receiving appropriate treatment among persons with STDR, the HBM provided us with the necessary structure and insights on strategies to improve decision making by families. Including the perceptions of HCPs contributed to a holistic understanding of the phenomenon of treatment seeking and served to triangulate our findings. The five components of the HBM model that predict an individual’s readiness to bring about behaviour change are described in Fig 1.

## Materials and methods

This qualitative component of the SMART India study (grant number MR/P027881/1) carried out using semi structured interviews (SSIs) complied with the declaration of Helsinki. It was approved by the Institutional Ethics Committees of all the participating institutions namely, Sankara Nethralaya Vision Research Foundation Institutional Review Board, Chennai, Tamil Nadu, Giridhar Eye Institute Ethics Committee Cochin, Kerala, LV Prasad Eye Institute Ethics Committee, Hyderabad, Telengana, Aditya Jyot Eye Hospital Ethics Committee, Mumbai, Maharashtra, LV Prasad Eye Institute Ethics Committee, Bhubaneshwar, Odisha, Asram Netra Niramay Niketan Institutional Review Board Haldia, West Bengal, Shri Aurobindo Medical Research Centre Institutional Review Board Raipur, Chhattisgarh and the Sri Sankaradeva Nethralaya Institutional Ethics Committee, Guwahati, Assam. Study details were explained to participants in the local vernacular following which written informed consent was obtained. The study period was from October 2020-August 2021.

### Sites

Of the 20 sites that had participated in the SMART India quantitative survey, we purposively selected 8 sites to provide some sense of geographical representation. The selected sites were: Chennai-state of Tamil Nadu, Cochin- state of Kerala and Hyderabad- state of Andhra Pradesh as three cities from southern India. Mumbai- state of Maharashtra from the west, Bhuvaneshwar- state of Orissa and Haldia- state of West Bengal from the east, Guwahati- state of Assam from the north east and Raipur- state of Chhattisgarh from central India. Each site was led by a locally based eye hospital with a trained ophthalmologist who was responsible for the study at that site.

### Sampling

Our participants sample included STDR patients who were referred to visit a health facility following screening. Those who attended the eye clinics were categorised as ‘sought care’ while those who did not attend despite being advised to do so were categorised as ‘not sought care’. From the SMART India survey, the research team prepared a list of those who had sought care and those who had not. Each patient on the list was contacted to determine their willingness to participate. Given our constraints of time and money, the mandate by our ethics committees to provide a sample size for their approval, our relatively narrow objectives (namely risk perceptions and barriers to seeking care) and the fact that an a-priori theoretical model (HBM) guided our study, we decided on a sample size of 32 patients who had not sought care (4 from each site) and 16 patients who had sought care (2 from each site). We were however, unable to achieve this number despite repeated calls and requests to the patients to participate in the study and were able to recruit only 11 patients from the not sought care group and nine patients from the sought care group. Inclusive of all 8 sites a total of 20 SSIs were carried out with persons with STDR. A combination of the COVID pandemic and the resultant lockdowns imposed by the central and state governments, poor phone and internet connectivity in some regions coupled with a reluctance to participate by some patients affected our ability to achieve the sample size that was originally planned.

The HCPs were ophthalmologists who treated persons with DR and were working in the eye clinics. The original plan was to do 16 interviews- two HCPs per site- but only 11 HCPs participated in the SSIs. This was because five HCPs (two each from the participating centres in Assam and West Bengal and one from Chhattisgarh) had relocated and were therefore not available to participate in the interview. All interviews with the HCPs were done at the respective facilities where they worked after obtaining written informed consent.

Separate interview guides (S1 Text) were developed for each of the stakeholder groups. The core elements were common for both patients and HCPs (i.e perceptions on susceptibility, severity, barriers to and benefits of care seeking). However, from the HCPs we also examined the roles they played in encouraging patients to seek regular care and sought their opinions on possible strategies that would aid better compliance.

### Data collection

The research teams who carried out the SSIs comprised optometrists who had a minimum of three years work experience and were employed as staff in the participating eye clinics in each of the study sites. In India, optometrists usually carry out all the initial assessments of the eye, interact closely with patients and serve as a link between the patient and the ophthalmologist. Prior to the recruitment of study participants an online training programme on qualitative research methods was conducted by SK and RM (both trained in qualitative research methods) over zoom sessions held for two hours every day over five days. The training sessions focused on orienting the field teams to the study methodology, the interview guides and to equipping them with the skills needed to carry out the SSIs. A combination of didactic lectures, demonstrations, mock interviews and hands on practical exercises were used towards enhancing the learning experiences of the research teams.

Interviews with the patients were done face to face with those who were willing to come to the hospital. Others who were unwilling to come on account of the risks posed by the COVID -19 pandemic were provided the option of an online/phone interview. Interviews with the HCPs were carried out at their respective eye clinics.

### Data management and analysis

All the audio recorded SSIs were transcribed verbatim and then translated into English (wherever necessary). The English transcripts were loaded into NVIVO. Data was analysed using a hybrid approach of qualitative thematic analysis [33] which incorporated i) a deductive a priori template of codes and themes derived from our research questions, the conceptual framework and the interview guides and ii)a data-driven inductive approach that was carried out following data collection. Given that the HBM constituted the basic theoretical framework that guided our study we put together a codebook (S2 Text) comprising categories/codes that reflected the HBM, namely, perceived susceptibility, perceived severity, perceived benefits, perceived barriers and cues to action. Following data collection, we used an inductive approach in data coding and applied the principles of thematic analysis as described by Clark and Braun [34], which involved six stages. The first was the data familiarisation stage which required us to become familiar with the data through repeated readings of the interview transcripts. We also began to note down our initial observations during this stage. Next was the process of coding. Three transcripts were initially coded independently by two coders (SK and RM). After independently coding 3 transcripts, we expanded the existing code book and added codes and categories inductively derived from the interviews. Any differences in coding were discussed and resolved. The remaining transcripts were coded using this code book, new issues identified in these interviews relevant to our study were given a new code and inserted into the code book and were organized according to the HBM categories. In the third stage we examined our coded data in the context of the HBM, looking to see how they fitted and whether it addressed our research questions. In the fourth stage we began to formalise our themes of analysis and to reflect on whether these themes actually related well to our data, were convincing and credible. In the fifth stage we labelled and defined each theme, describing in detail what it signified and in the 6^th^ stage of the analytic process we brought forth a coherent explanation of our study findings. Sifting through the data we then sorted and selected quotes and placed them under appropriate themes.

## Results

### Socio-demographic characteristics of participants

Of the nine patients from the sought care group, three were men and six were women while the 11 participants from the not sought care group comprised two men and nine women. Those from the sought care group were a little older with a mean age of 59.8 years (Std ±9.19) while participants from the not sought care group had a mean age of 53 years (Std ±6.60). Five participants from the sought care group had completed five years of schooling (primary school), three were non-literate and one was a graduate. In the not sought care group, six participants had completed five years of schooling; three had completed nine years of schooling (secondary school) and two were non–literate. All participants were married; the women were all homemakers while the occupations of the men ranged from casual labourers, tailors to running a petty business. The 11 HCPs included five men and six women, all were consultant ophthalmologists. Seven of them were retinal specialists.

### Themes of analysis

We mapped the care seeking journey of patients with STDR guided by the HBM and present four themes that we believe best explained our data i) Understanding of STDR and its treatment ii) Perceptions about susceptibility and severity (Perceived threat) iii) Perceived barriers iv) Perceived benefits and cues to action.

#### i) Understanding of STDR and its treatment

Perceptions about susceptibility to a particular disease/illness are considerably influenced by the patient’s understanding and awareness of the disease, its symptoms and whether they believe it could seriously impair their health. We explored understanding of STDR from the point of view of those patients who had sought care and those who had not and triangulated this with the opinions of the HCPs.

*Sought care group*. Patients who had sought care indicated some awareness about STDR when they reported having been told that the disease could affect their vision perhaps even resulting in loss of sight. As described by this participant from Bhuvaneshwar,

“*When I was ill I have visited the government hospital*, *after check-up I came to know that I have diabetes*. *Doctor told me that it is not that only my health will be affected but my eyes can also be affected*. *I have consulted an eye doctor and the doctor informed that in many cases one may even lose vision”*,

(Bhuvaneshwar-sought care-02, Female, 65 years).

Information about STDR had been communicated to them in some cases, by the doctor who had been treating them for their diabetes and in other cases by the eye specialists to whom they had been referred. One patient described STDR as, “*bleeding in the retina”*, while another spoke of the risk of a person dying when she said,

“*When the sugar reaches into the brain then person may die or eye also may get affected*. *These are not new things*, *such general information must be known by all of us”*,

(Chennai-sought care-01, Female, 35 years).

Thus, patients who had sought care professed to not having heard about DR in the beginning but became cognizant of it over the years when their doctors told them about it and advised them to visit an eye specialist. They described being told about the importance of controlling their blood sugars and realised that if this control was not adequate it could affect their eyes leading to possible loss of vision. One participant from Guwahati said,

*“The doctor informed me that it is the beginning of Diabetic Retinopathy*. *I did not know earlier but*, *after the doctor explained I know a little better now*. *I have been suffering from diabetes for the last 10 years but I never knew it can also affect the eyes*. *…The doctor also informed me that this is a sight threatening disease and can lead to complete blindness at any time*, *without my knowing*. *I am well aware now and I have also begun the treatment for it”*,

(Guwahati-sought care-01, Male, 47 years).

They were also aware that treatment for STDR involved injections and/or laser treatment. Undergoing regular eye check-up, either once in 6 months or once every year, was therefore considered very important.

The HCPs believed that STDR was a serious problem and it was critical that it be detected early so appropriate treatment could be started. Failing this, they felt sceptical of the patient recovering vision despite best efforts. Patients were believed to have poor understanding of the importance of consistent control of blood sugars as highlighted by this HCP from Chennai, who said,

“…*they tend to think that if one day they have good control* [of food intake] *then it should reflect on them the very next day or improve their vision*….. *So we have to explain to them and make them understand that diabetic retinopathy when it reaches the stage of severity we can probably only stabilize the disease*, *we cannot improve too much or reverse the complications of the disease completely”*,

(Chennai-HCP-02, Male, 38 years).

Doctors from Haldia and Raipur said that overall awareness about diabetes and its complications was very low among rural and tribal populations. The HCP from Raipur said,

“*The problem is in the villages*, *patients of advanced diabetic retinopathy come from there because there is no one to investigate and due to lack of information about the disease*, *there is more problem”*,

(Raipur-HCP-01, Male, 40 years).

Further, he added that in rural areas testing facilities for diabetes were not well developed and hence patients lost out on early detection. The doctor from Cochin said that sometimes diabetes was either not diagnosed or people had not started treatment for diabetes with oral hypoglycaemic agents. He also said that some individuals took alternative medicines for diabetes that did not help in glycaemic control. Such patients would invariably come at an advanced stage of the disease.

*Not sought care group*. A mixed picture emerged with respect to those who had not sought care or who had not returned to the hospital for a follow up, with some being quite unaware and others being familiar with STDR. Those who had not heard of STDR or of its possible effects on the eyes had been seeking treatment for management of their diabetes but had never gone for an eye check-up. They also said that their health care providers had not advised them to do so. One participant even questioned the need to go for an eye check-up and said,

“*I don’t need to go for an eye check-up*, *I am able to see and do my daily routine and all is ok*.. *After diabetes only changes made are in my food*, *nothing else and I am seeing everything as usual*… *you said something about diabetic retinopathy*... *I don’t know about this and have never checked up for this*. *I have gone to the village health camp*, *I check up there and they didn’t say anything about this*.*… no one informed me for first time I am hearing from you”*,

(Bhuvaneshwar-not sought care 01-Female, 61 years).

Some HCPs too, blamed the physician/diabetologist of these patients as they had failed to educate them adequately. As reported by one of them from Haldia,

*““*… ., *most of them* (patients)*say that they were not aware that diabetes can cause vision loss*, *nor the treating physician has educated them about it and asked them to get their eyes checked*. *I think the treating physician should educate every patient about diabetic retinopathy and should ask them to get their eyesight checked with an Ophthalmologist”*,

(Haldia-HCP-01, Male, 37 years).

Other HCPs said that patients, despite being advised by their doctors did not go for regular eye tests

“*…some patients despite telling them multiple times they don’t lend the ear*. *They feel that the eye treatment is different and the diabetic control is different*. *So they have to understand that the glycaemic control*, *the blood sugar control is very important along with the treatment for diabetic retinopathy*… *the awareness is lacking*”,

(Chennai-HCP- 01, Female, 38 years).

All the HCPs felt that treating physicians played an important part in educating patients about DR and STDR.

The group who were aware of STDR, yet had not sought care, appeared to be cognizant of the possible harms to their health in terms of vision loss brought on by their diabetic condition. Their doctors had explained to them in fair detail about the importance of regular visits to the eye hospital and of keeping their blood sugars under control. However, as they were not experiencing any symptoms they did not believe anything was wrong,

*“I only have vision problem in eye since 6 to 7 years that too after I was diagnosed with diabetes*. *I am not taking any medicines for my eyes till now*….. *I do not see any changes*. *Everything seems normal to me…only thing is it looks dull and is not bright”*,

(Chennai- not sought care-01, Female, 56 years).

Another participant despite repeated reminders did not perceive any urgency to seek care and said,

“*I was advised to do an eye check when I came to the hospital last year…but then corona came and I came to the village during this period*. *I used to get calls from the hospital asking me to come for a check-up*. *I told them that I will come when I come to Mumbai”*,

(Mumbai-not sought care-01, Male, 55 years)

The HCPs agreed that the slow progression of STDR combined with the virtual absence of symptoms deluded patients into thinking that they were fine. Patients were unaware that diabetes could in fact affect their vision. In this context, one HCP from Haldia declared that many patients would perhaps seek care only when symptoms become debilitating.

“*The main problem with diabetic retinopathy is that the patients’ eyesight is normal in the earliest stage which causes delay in seeking treatment*. *Only when they develop macular oedema or vitreous haemorrhage they seek treatment which by then has become sight threatening”*,

(Haldia-HCP-01,Male, 37 years).

Endorsing this lack of understanding of the disease, another HCP said,

*“I think that it is really serious problem because most of our population don’t know about how seriously sight threatening diabetic retinopathy can be*… *they don’t understand that diabetic retinopathy or diabetes itself is a very morbid disease*. *They feel that if they take medication*, *it can get controlled and so there is nothing to be worried about”*,

(Mumbai-HCP*-*02, Female, 28 years).

#### ii) Perceptions about susceptibility and severity (Perceived threat)

Given the apparent lack of threat of a possible loss of vision consequent to a diagnosis of STDR- evident from the reports of many patients and validated by the HCPs- we explored threat perceptions as perceived by both the ‘sought care’ and ‘not sought care groups’. Important to note is the fact that both groups had their share of dissenting voices. Here too we have triangulated these perceptions with the reports of the HCPs.

*Sought care group*. Typically, participants who had sought care were perceptive to the threat of possible vision deterioration/loss consequent to the diagnosis of STDR and to that extent were sensitive to the need to be consistent in the care of their eyes,

*“Yes I know about diabetic retinopathy*, *When I visited the government run PHC* (Primary health Centre) *the doctor explained to me about diabetic eye disease and informed that in many cases one may lose one’s vision*…*; I am taking medicines for it*, *doing exercise and regular eye check-up because diabetes can affect my eyes*, *many people ignore them*, *we should check our eyes regularly and take care*”,

(Bhuvaneshwar-sought care-01, Female, 67 years).

Such patients tended to take necessary precautions to safeguard their eyes. However, even among the sought care group there were a few who appeared unconvinced–the dissenting voice as it were—about the potential risk of vision loss, as this patient who said,

“*I don’t know if sugar patients* (diabetes is colloquially referred to as ‘sugar disease’) *might lose vision without any pain or symptoms*. *I am not having any pain*. *My distance vision only is bad*, *near vision is good*. *I do not know if sugar patients should visit an eye doctor once in a year*. *I can see well*. *I feel this is not going to affect my eyes because my blood sugar is normal”*,

(Hyderabad-sought care-02,Female, 74years).

The absence of symptoms was thus an overarching feature that lulled patients into a false sense of security.

The fact that patients did not perceive the seriousness of their illness in the absence of any symptoms was also highlighted by the HCPs. One of them from Mumbai said,

“… *for them* (referring to patients) *this is not important as it has not affected them yet*. *It is only when the disease starts to affect them that they become compliant or that they become responsive*. *… They don’t understand the seriousness of the situation*,*”*

(Mumbai-HCP-02, Female, 28years).

She went on to say that patients may continue to ignore the retinopathy when it is in its early stages little realising the rapidity with which deterioration could set in.

“*even if they develop a mild form of diabetic retinopathy*, *it will progress rapidly because they don’t know that they need to get checked up… they don’t have the knowledge*. *So*, *I think the slum population*, *urban*… *you know below poverty line kind of population*, *uneducated people are the ones I feel who are most at risk”*,

(Mumbai-HCP-02, Female, 28 years).

*Not sought care group*. As regards the ‘not sought care group’ while poor risk perceptions attributable to some extent to inadequate understanding of the disease had a bearing on their failure to seek care, there were a few who were different. They were different in that a sense of fear of the possibility of losing vision had begun to permeate their consciousness. One such participant voiced his apprehensions and said,

“*I’m worried whether my problem will be cured*. *Will I lose my eyesight as informed by your health worker*? *I never knew such an eye disease even existed*. *Having high sugar is such a problem…*- *Initially I was not serious at all*, *as I did not know much about it*. *But when I was informed by the health worker that it might lead to sudden complete blindness I got very afraid*. *The scariest part is that such a serious issue was happening in my eye and I wasn’t even aware about it*.—*I did not know earlier that diabetes can lead to loss of eyesight”*,

(Guwahati-not sought care-01, Female, 40years).

Further throwing light on patients’ care seeking behaviours, the HCPs said that patients tended to attribute the deterioration of their vision to cataract which was not considered a threatening condition and one which could be rectified with surgery. This contributed to reduced threat perceptions and to delays in seeking care. The situation was compounded by the fact that many patients either remained unaware about their diabetic status or else were not consistent in maintaining their blood sugars. Highlighting this, an HCP from Bhuvaneshwar said,

“*Longer the years with diabetes greater are the chances of developing DR*. *Poor glycemic control*, *association of hypertension and dyslipidemia may raise the chances of vision loss*. *Renal disease*, *high body mass index*, *smoking*, *alcohol and poor health seeking behaviour may lead to early progression of the disease”*,

(Bhuvaneshwar-HCP-01, Male, 48 years).

#### iii) Perceived barriers

We next explored specific barriers that impeded care seeking among patients with STDR. Our analysis revealed that similar barriers were reported by patients from both groups and therefore we present our findings under four sub-themes, i) Cost of care, access to care and financial constraints ii) Covid and the lockdown iii) Support iv) Gender related issues. The last sub-theme on gender related issues reflects the opinions of the HCPs alone.

*Cost of care*, *access to care and financial constraints*. The predominant barrier reported by patients across both groups was the high cost of treatment for STDR. Patients advised monthly intra-vitreal injections found it difficult to bear the high cost of these injections (there are many types of such injections ranging in cost from around Rs.10,000 to Rs. 100,000)which severely compromised their ability to seek care. Furthermore, the progress of treatment was slow and required repeated visits which not only added to their costs but was deeply frustrating. As reported by this patient,

“*Economic reason is the main reason for not coming early…*. *As the treatment of my disease is very costly and takes time it is unaffordable by people like us…I am in a lot of tension as my right eye has a lot of problems and I need urgent treatment*. *Eleven months ago the doctor told me to come… it is already late and now I am almost going blind”*,

(Haldia -not sought care-02, Male, 51 years).

For patients from rural and remote areas, accessing care facilities posed a major challenge as these were usually located in cities and towns, far away from their homes,

“*I am worried that I won’t be able to do the treatment because this is the only eye hospital in the district and my place is around hundred kilometres from here*. *I have also heard that the injections that I need is also very costly and I might not able to afford the treatment at all”*,

(Haldia-not sought care-03, Female, 49 years).

Difficulty in taking time off from work was another barrier. A visit to the hospital meant a whole day off from work which poor patients could ill afford,

“*Yes*, *actually I won’t get time*. *I work in a bungalow* [does household work], *so doing work there itself occupies my time*. *I was told to spend a day to consult in an eye hospital*, *but I didn’t get time*, *I left just like that”*,

(Chennai-not sought care-02, Female, 56 years).

The HCPs admitted that the management of the disease was expensive, complex and required regular monitoring. These were major deterrents to seeking care for patients. Those without the benefit of any health insurance cover, mainly the daily wage earners were particularly hard hit. Neither could they afford the treatment costs, nor could they afford to take a day off from work to visit the hospital as this would mean loss of wages. Such patients they said tended to drop out early. For other patients the fact that they needed to continuously monitor their health, deal with other diabetic related complications coupled with the realization that this would be a life-long feature was very exhausting. As one doctor from Mumbai explained,

*“Patients sometimes do lose faith*… *they tend to drop out either because they are financially unable to afford it or because they think it is not being cured or they think it should happen faster*. *Sometimes the other systemic diseases are so severe that they are not able to come for treatment of their eyes”*,

(Mumbai-HCP-01,Female, 34 years).

*Covid and the lockdown*. The lockdown imposed due to the COVID-19 pandemic was another reason cited by a few patients that came in the way of their care and treatment. One patient from Mumbai who did not seek care said that her family members forbade her to seek treatment for her eyes because of the lockdown and the added fear of her contracting the COVID-19 infection. A patient from Haldia regretted having delayed seeking care and with the lockdown in force, feared the effect this additional delay would have on her eye sight. She said,

“*I couldn’t come due to lockdown and monetary problems*. *I wish I had listened to my doctor and not waited for so long to get my eyes checked*. *Maybe then my problem would not have been so bad”*,

(Haldia-not sought care-03, Female, 49 years).

A patient from Cochin describing the difficulties she faced in accessing the eye hospital said,

“*I am living in a rural area the mode of transportation is by boat and bus*. *Due to the pandemic the bus has stopped the service and the boats are limited which makes it really difficult for us to travel to the eye hospital*, *especially because of its timing”*,

(Cochin-not sought care-01, Female, 62 years).

Patients living in rural areas reported that the government usually organized free eye camps periodically which gave them an opportunity to get their eyes tested. The camps did not happen due to the lockdown which was a major blow to the village folk.

All the HCPs were unanimous in their opinion that the Covid pandemic and the resultant lockdowns were a major setback to patients as many were unable to obtain the care they needed.

*Support*. The need for assistance, be it from a family member or a friend to accompany them to the hospital for a consultation or a follow–up visit was expressed by several patients. The problem was exacerbated for those who had to travel long distances to access care. Coupled with their impaired vision, negotiating the challenges of boarding buses and trains on their own, severely affected their ability and desire to seek care, as reflected in this quote from a participant from Cochin,

“*Actually I am having blurred vision since 1 year and I consulted in a hospital at Ernakulum and they told me to consult at a higher center*. *I have no one to accompany me and also considering my age I was unable to go”*,

(Cochin-not sought care-01, Female,61 years).

The HCPs reiterated the need for good family support. They stated that a large number of such patients were older in age and some also suffered from low vision. One HCP from Cochin added that diabetes and STDR required lifelong care for the patient. This necessitated that patients have the support of their families without which their care and recovery would be compromised,

“*…one important barrier is it requires life-long care which is not easy because patient will be having multiple diseases they have to go to the diabetologist*, *and if they have nephropathy*, *they have to go to nephrologist and the ophthalmologist is one among them*… *it is not that they come once*, *they get treatment and it is over*, *they have to keep coming again andagain*.. *they need family support*. *Most of the patients are generally middle aged or elderly and with poor vision they won’t be able to come alone”*,

(Cochin-HCP-02, Female, 51 years).

*Gender related issues*. With respect to gender, a few HCPs were of the opinion that men sought care early when compared to women. Explaining this, the HCP from Chennai said that often women tended to come when symptoms became very debilitating, an indication that the disease had progressed to an advanced stage,

“*The females are probably more delayed because even after a significant drop in vision*, *they do not reach because they are mainly at the household*, *so they don’t reach us*. *Males*, *reach a little early because they are working population and it affects their work”*,

(Chennai-HCP-01, Female, 38 years).

Others spoke of these differences in care seeking as more evident in rural areas and said,

*“The patriarchal nature of society here appears to be a barrier to the females*. *Also most females do not want to be a burden on the family*. *So somewhere health becomes less of a priority for the females”*,

(Haldia-HCP- 02, Female, 34 years).

Another doctor, also from Haldia believed that there were many cases of undiagnosed diabetes among women, primarily because these women were uneducated, totally dependent on their husbands/families and did not feel comfortable speaking of their diabetes or eye problems to their in-laws. Care seeking among these women was therefore poor.

“*Females are mostly dependent on the caregivers*, *that is the husband and offspring with regards to their health check-up…many of them are housewives and not very educated*. *Even younger females with DR present late and mostly the reason I see is they are not very comfortable speaking of their poor vision and about their diabetes to their family members/in laws*. *There are lot of undiagnosed diabetes in females with DR”*,

(Haldia-HCP-02, Female, 34 years).

#### iv) Benefits and cues to action

Our final theme sought to understand from patients whether they perceived any benefits in seeking care and in bringing about lifestyle changes and what cues would help sustain these behaviours. We also explored the concept of “cues to action” from the perspectives of the HCPs. We believed that by virtue of their years of experience treating such patients they would be in a position to provide insights into what could help improve care seeking in these patients.

*Benefits*. Fear of poor health because of diabetes and of the potential of losing vision, was a strong motivating factor that pushed patients into seeking care and to following the doctor’s recommendations. If this meant going for regular eye check-up and keeping blood sugars under control through proper medication and lifestyle changes, then these patients felt this was worth it. As stated by a patient,

*“As much as possible I take medicine prescribed by doctor*. *I feel better after medication and also my sugar level is in control*.. *I have diabetes*, *regular eye check-up is necessary for me so I can protect my eyes*”,

(Bhuvaneshwar-sought care-02, Female, 65 years).

Another patient feared becoming helpless and dependant on others and said,

*“I will not be able to take a glass of water*… . *our children do not stay with us*.. *No one stays nearby*.. *We*, *husband and wife are alone*.. *my husband will go out for work and I will be alone in the house*.. *If I will not be able to see properly then who will look after me*.. *How will I manage without my eyes*.. *So we came to the hospital for treatment”*,

(Mumbai- sought care-01, Female, 58 years).

Even those who had not sought care, in hind sight, regretted their actions and understood the damage this had done to them,

“*I fear that I might lose my sight completely*, *I cannot cook food for myself*. *I have to depend on my family members a lot*. *I used to do all the work myself before*, *now it is not possible*. *I wish I had come earlier to the hospital”*,

(Haldia-not sought care-01, Female, 59 years).

Thus, many patients appreciated the benefits of regular eye check-up and care of the eyes but had been hindered from accessing care on account of the barriers described earlier. Therefore, the cues to action recommended by both patients and HCPs are primarily aimed at addressing these barriers.

*Cues*. Several cues to action were reported by both patients and HCPs which we categorized as improving awareness, improving access to and reducing costs of care and need for support.

*Improving awareness*. The need for making the public and in particular, persons with diabetes more aware about the disease was considered important to promoting better care seeking by many patients,

“*I think it is extremely important to create awareness among diabetic people regarding DR*. *All diabetes treatment centres should hold awareness camps on DR from time to time*. *In your hospital you can display videos on DR informing diabetic patients to do a proper retina check-up at least once a year*. *You can also appoint health workers to increase awareness in the communities”*,

(Guwahati-sought care-01,Male, 47 years).

Those who had not sought care highlighted that it would be helpful if the treating physicians advised and provided proper guidance,

*“my doctor said to me “your eyes are normal only”*. *He said there is no problem in my eyes due to diabetes…If there is problem in the eye then he would have told me right*? *He said it’s normal so I left it like that”*,

(Chennai-not sought care-01, Female, 56 yrs).

Sending regular reminders to patients to come for an eye check-up and warning them of possible repercussions of not seeking treatment for STDR was also seen as a way to both educate and get patients seek care.

The HCPs believed that poor awareness about STDR constituted a major challenge to care seeking among patients and saw this as a critical first step to encouraging patients to seek proper and timely treatment for STDR. They believed that the poor and less literate patients were particularly hard hit and often failed to appreciate what they were up against,

“*Poor patients and those with low literacy may fail to understand the importance of regular follow up*. *Well informed patients however participate in the treatment better*…*”*,

(Bhuvaneshwar-HCP-01, Male, 48 years).

According to them this was where the treating physicians—generally the first point of contact for most patients- could play a significant role. In this context one HCP said,

“*The physician should tell the patient that because you are diabetic you need an eye check-up*, *you have to go to ophthalmologist*, *you should take treatment on time*… *that guidance if it comes from their family physician that will have a good influence on patients and they will tend to obey…I think these physicians should play a more important role”*,

(Cochin-HCP-02, Female, 51 years).

Another HCP spoke about the value of involving the community health workers in spreading awareness about DR and STDR to people in the communities in which they lived. Displaying messages on the print and social media as a way of publicly disseminating information on STDR was also reported. In addition to educating patients on STDR, HCPs also stressed the need for counselling and motivating patients to cope positively and more effectively with the disease. An HCP from Chennai described using words of encouragement and appreciation with his patients and said,

*“So every time I see patients*, *I tell them*, *“you are doing good and your blood sugar is under control*. *I have to keep seeing you regularly then only you will be maintaining the same good vision*. *So*, *positive reinforcement does work”*,

(Chennai-HCP- 02,Male,38 years).

*Improving access to care and reducing costs*. For patients from rural and other remote areas having hospitals located closer to their villages and towns was believed to both motivate them to seek care and enable easy access. In this context one woman participant from Haldia suggested,

“*My home is very far away*. *It takes 3–4 hours to reach here and also costs a lot of money to come here*. *So it would not be possible to come here for check-up regularly*. *It will be helpful if we could go for follow-up to any nearby eye hospital as it is not possible to come here all the way for regular follow-up”*,

(Haldia-not sought care-03, Female, 49 years).

Conducting free eye camps in their villages and in nearby towns were also suggested as helpful strategies that would make care accessible and affordable

The HCPs admitted that treatment for STDR was expensive and involved injections or laser or surgery or a combination of them. Describing this, an HCP said,

“*Treatment mainly depends on stage of disease in which the patient presents to you*. *Early stages of DR can be managed with good glycaemic control and control of associated risk factors*. *But in later stages of disease progression intra-vitreal injections and lasers are the mainstay of treatment”*,

(Bhuvaneshwar-HCP-02, Male, 33years).

Therefore, seeking early and regular care was strongly suggested. Another suggestion to improve both access and reduce cost for patients was for the government to step in and equip government hospitals (where care is provided free) with the capability to deliver such care,

“*The government should take active role in training all their primary healthcare providers on the importance of eye check-up in treatment of diabetes*”,

(Haldia-HCP-02, Female, 34 years).

Reducing the cost of the intra-vitreal injections and making them available in the government hospitals were recommended. The HCPs also highlighted the existence of various government health insurance schemes which patients need to be made aware of and which could help to subsidise the cost of care.

*Support*. Support in terms of having someone to accompany them to the hospital was seen as an important factor that would aid better compliance with hospital visits. In many cases both patients and their primary caregivers, usually the spouse were elderly, making travel to the hospital a challenge for both. Younger family members like their children or other relatives who were called upon to take the patient to the hospital were often constrained by their own work and home responsibilities. Therefore, one suggestion was to have the hospital organize a vehicle to pick up patients from their homes for a check up on specified days. As reported by this patient,

“*The hospital can arrange van for patients to travel*. *Prior to travel they can send a reminder and inform patients that we have arranged van for travelling…So trip can be organized to pick up and drop the patients*. *Like these if you inform*, *people will show interest to come*, *we poor people will expect these things only…so if hospital provide such services it will be helpful to us”*,

(Chennai-sought care-01, Female, 35 years)

The HCPs highlighted that STDR was a slow progressive condition, and the treatment too was long drawn and expensive. In view of this, they felt that the support of family members to motivate and encourage patients to maintain a healthy lifestyle, assist with financial costs and accompany patients to the hospital whenever required were critical cues to the patient’s well-being and to improved care seeking as well. In this context an HCP said,

“*It is the family who keeps on motivating when the patient loses hope as treatment for DR is a prolonged one*. *Just treatment of the eye alone is not enough*. *It’s the holistic improvement in the patient’s blood sugar control other systemic factors and most important the stress levels*. *This is where family support plays an important role*. *Most diabetic patients are aged and need the help of their family*…*”*,

(Haldia-HCP-02, Female, 34 years).

## Discussion

The findings from our study revealed poor appreciation of the need for early and sustained care among most patients with STDR. Added to this, the absence of symptoms and the slow progression of the disease contributed to low risk perceptions which further compromised seeking early care. Barriers in terms of prohibitive costs of treatment, difficulties in accessing care services and inadequate social support complicated the scenario by increasing health disparities.

Low awareness contributed considerably to poor care seeking and perceptions of risk, a fact that is well acknowledged in low and middle income countries such as India [35–40]. There are studies showing that those with less education who usually belong to the poorer sections of society tend to have poor knowledge about DR and its complications [41]. By virtue of this fact they tend not to perceive the seriousness of the situation [25] and therefore avoid or delay care seeking.

In our study, although most patients who had sought care were aware that diabetes could affect their vision there were also those who did not appreciate this fact. They nurtured the belief that as they were asymptomatic and had no vision related difficulties, they were fine. To them having diabetes essentially meant controlling diet, exercising and taking the required medication. An earlier study carried out in rural Tamil Nadu [38] found that while 90% of the study participants were aware of the importance of regular eye examinations, around one-third believed that if blood sugars were controlled there was no requirement to see an ophthalmologist. The study further highlighted that despite having knowledge about DR, only half the study participants, knew about the effects of good control of diabetes on DR. The HCPs in our study also emphasized that significant numbers of patients did not recognize the importance of consistent control of blood sugars and added that in many cases the treating physicians of these patients did not advise them to go for an eye test. Previous studies have shown that duration of diabetes, poor glycaemic and blood pressure control and dyslipidemia [42, 43] accelerate the development and progression of DR suggesting the need for more intensive management of the condition which can be overwhelming to patients. Given this, enhancing understanding and awareness about STDR would perhaps be a key priority to address to encourage better compliance and help prevent vision loss.

What was particularly alarming in our study was that despite all reminders to attend the eye hospital and the offer of free screening, patients did not seek care. In an earlier study [25], we had recommended giving due credence to a patients’ understanding capacity when providing health care awareness as a means to enhance better learning and thereby to improving treatment compliance. As stated by Piyasena et al. [40]*“an individual’s better understanding of their susceptibility to vision loss may increase motivation to attend a screening examination”*. Perhaps, involving their treating physicians/diabetologists- with whom patients share a relationship of trust—in this effort could be a step in this direction. In this context, Sanghamitra et al. [44] have spoken about the need to strengthen the capacity of physicians in the private sector to counsel patients about the importance of seeking appropriate care, thereby enabling patient recovery. Equally important will be the need to create more patient friendly environments making the entire testing and treatment process less frightening and intimidating to patients.

Further, the insidious progression of STDR contributes to a false sense of security increasing the perceptions that regular eye tests are unnecessary [45, 46]. In this context, Xiong et al. [36] reported that presence of symptoms can serve as triggers which could alert patients to the need to seek care. Understandably, this does not apply to DR as visual impairment occurs late in the disease and is usually associated with sight threatening complications such as diabetic macular oedema and complications of proliferative diabetic retinopathy. Devenney and O Neill [47] described the sense of loss of independence and a variety of social losses faced by DR patients with fading vision which could serve as possible cues to action. However, by then the disease would have progressed significantly with potential for irreversible visual loss. Therefore, educating patients with diabetes about DR would be most essential for informed decision making and appropriate care seeking.

In our study we found that where women were concerned, not only was awareness poor but women refrained from reporting their problems to their family members as they felt awkward or else did not want to pose a burden. Studies have shown [40] that women-many of whom are dependants and have no income of their own -are reluctant to seek eye care and unwilling to draw on the limited family resources for their treatment. Das et al. [48] described that women participants in their study felt that men tended not to consider women’s health problems as serious and often believed that they were faking it. Greenwood et al. [49] explored similarities and differences in values and attitudes towards women in the work place and at home across several countries in Asia. They reported that, *“culturally ingrained gender role expectations keep women from full equality at work and at home”*. Given this climate, women may lack both the voice and the economic capability to seek care for themselves. The need to sensitise families and men in particular to be attentive to and supportive of the health needs of women members in their family would be extremely important.

The prohibitive cost of treatment was another impediment to seeking/continuing treatment. Financial barriers to DR treatment is a universal problem in both developed and developing countries [39, 50, 51] attesting to the urgent need to address this issue. Our study reported that patients who were poor or who lived in rural/remote areas were particularly affected. The HCPs confirmed that it was challenging for patients on monthly intra-vitreal injections to bear the high cost of these injections in the absence of any insurance cover, often leading to non-adherence to treatment regimens. Suggestions towards making these injections available in government facilities at reduced cost and informing patients about the existence of various government health insurance schemes were made by both patients and HCPs in our study. In this context the government of India under the NPCDCS (National Programme for Prevention and Control of Cancer, Diabetes, Cardiovascular disease and Stroke-2013-17) [52] has focused on strengthening infrastructure, human resources, early diagnosis, management and referral for non–communicable diseases such as diabetes. Provision has also been made for free diagnostic facilities and drugs for patients attending these clinics. Incorporating screening and treatment for DR and STDR under this programme will facilitate a larger proportion of patients to avail and access these services at more reasonable costs.

Poor support in terms of not having any one to accompany them to the hospital was another major challenge reported by participants in our study. Sanghamitra et al. [44] underscored the importance of family and friends who were seen as enablers in terms of providing encouragement and motivation to patients to manage their diabetes. Studies have shown that presence of such support improves diabetes control, knowledge, and psychosocial functioning [53]. We could perhaps also draw upon the strengths of the community health workers usually attached to the government run primary health centres (PHCs) located in villages and urban communities who by virtue of the outreach work they do, are in close contact with the communities they serve. They could be trained to both educate and support men and women to more easily access eye care services.

### Strengths and limitations

This study used the HBM with its focus on intra-personal factors, including risk-related beliefs which influence decision making as a means to understand the phenomena of care seeking for DR. Using a hybrid approach of qualitative thematic analysis involving a combination of deductive and inductive strategies is the strength of this study. At the outset, the use of the HBM provided logic and structure to the study and also allowed for a data-driven inductive approach that was carried out following data collection.

We were however, unable to achieve the desired sample size which could be seen as a limitation. We compensated for this lack by taking care to explore each domain of the HBM as thoroughly as possible with each respondent during the interviews. At the stage of coding we kept our minds open to the generation of new codes and categories beyond the ones developed a priori thereby helping to further saturate each domain of the HBM. It is important to highlight here that our patients, albeit from different cities across India, were all persons with diabetes, aged between 40 to 60 years and predominantly belonging to low and middle class socioeconomic backgrounds. Further, across different states in India, issues concerning awareness of, access to and compliance with care for DR were found to be similar in this study. While we did not undertake any comparison of data between states on account of the very small sample sizes in each state, the fact remains that patients and health care providers across all the states raised similar issues thereby enhancing the transferability of these findings to poor and socioeconomically deprived populations in our country.

## Conclusion

The findings from this study add to the growing body of literature from different countries attesting to the urgent need for greater health literacy around diabetes, DR and STDR. Undoubtedly, a real understanding of the disease was lacking among patients in our study evidenced by the fact that many did not appreciate the effects of good control of diabetes on DR. While recommendations to enhance health literacy around DR have been repeatedly made [38, 54], it is unfortunate that this continues to be a major issue severely compromising care. Given its irreversible nature eventually leading to vision loss if left untreated, a redoubling of efforts towards improving patient awareness and compliance will be vital to ensuring a better quality of life for DR patients.

## Supporting information

S1 Text Study guides. (DOCX)

S2 Text Study code book. (DOCX)
