# Supplementary material for: ‘I don`t need an eye check-up’. A qualitative study using a behavioural model to understand treatment-seeking behaviour of patients with sight threatening diabetic retinopathy (STDR) in India
Source: PLoS One. 2023 Jun 15;18(6):e0270562. doi: 10.1371/journal.pone.0270562 (PMC10270603; doi:10.1371/journal.pone.0270562)
Supplement: S1 Text — (DOCX) [file pone.0270562.s001.docx]

**Qualitative Component- SMART India Study**

**SSI with STDR patients who did not seek care**

1. Can you please describe to me what you believe is the problem with respect to your eyes? *(Probe : what respondents think are the condition they are suffering from, how do they refer to it, what do they think could happen to them because of this problem, how has their life changed because of this problem)*
2. How serious a problem do you think this is and why do you feel so? *(Probe: respondents feelings of fear, of possible loss of vision , what do they think they need to do, do they feel they need to go to hospital to seek treatment, have they gone before or are irregular)*
3. Was any advice given by any health care provider regarding what needs to be done for the management of the eye condition, if so what was the nature of advice given and did you follow it? *(Probe: respondents perception of the usefulness or benefit of the advice given, do they believe it to be helpful or not and reasons for this)*
4. Are they any concerns or worries that you have in following the advice given to you by the health care providers, please describe what these are? *(Probe: allow respondents to describe any barriers or difficulties they perceive including financial, personal, accessibility to health facility, poor social/familial support etc)*
5. Can you please describe what would be helpful to you in enabling you to seek care for treatment of your eyes? *(Probe : for eg presence of someone to encourage and motivate them, talking to others with the same problem, more details and information about the health condition etc*)
6. Are you aware that diabetes can cause blindness without a patient having any symptoms? (*Probe: whether they think that people with diabetes should have their eyes checked for diabetic changes every year)*

**Qualitative Component- SMART India Study**

**SSI with STDR patients who sought care**

1. Can you please describe to me what you believe is the problem with respect to your eyes? *(Probe : what respondents think are the condition they are suffering from, how do they refer to it, what do they think could happen to them because of this problem its implications for their daily life )*
2. How serious a problem do you think this is and why do you feel so? *(Probe: respondents feelings of fear, of possible loss of vision, what do they think they need to do, do they feel they need to go to hospital to seek treatment)*
3. Was any advice given by any health care provider regarding what needs to be done for the management of the eye problem, if so what was the nature of advice given and are you following it?did you follow it? *(Probe: respondents perception of the usefulness or benefit of the advice given, do they believe it to be helpful or not and reasons for this)*
4. Are they any concerns or worries that you have in following the advice given to you by the health care providers, please describe what these are? *(Probe: allow respondents to describe any barriers or difficulties they perceive including financial, personal, accessibility to health facility, poor social/familial support etc; how many times in a year is recommended for review, are they aware of the signs of deterioration )*
5. How confident do you feel about being able to follow the recommendations given you?
6. Can you please describe what was particularly helpful to you in enabling you to seek care for treatment of your eyes? *(Probe : for eg presence of someone to encourage and motivate them, talking to others with the same problem, took efforts to understand more details and information about the health condition etc*)
7. What do you believe are the reasons why some people with STDR do not seek care and what do think can be done to encourage them to seek care? *(Probe: seek their suggestions and ideas on what will be helpful)*
8. Are you aware that diabetes can cause blindness without a patient having any symptoms? (*Probe: whether they think that people with diabetes should have their eyes checked for diabetic changes every year)*

**Qualitative Component- SMART India Study**

**SSI guide for HCPs**

1. How serious a problem do you think STDR is and why do you feel so? *(Probe: respondents understanding of the condition, its prevalence, how many cases usually seen, gender issues, who are at risk)*
2. What is the nature of treatment provided for STDR? *(Probe: nature of treatment usually given, effectiveness of treatment)*
3. What is your perception about awareness about STDR among diabetic patients and on what do you base this?
4. How compliant do you think patients generally are with regards to any advise or recommendations you give them? *(Probe: whether diabetic patients come for check up as advised, perceived barriers faced by them, perceptions on reasons for non-compliance and/or good compliance)*
5. What do you think are the reasons which makes some patients compliant and others non compliant? *(Probe : for eg presence of someone to encourage and motivate them, talking to others with the same problem, more details and information about the health condition being sought, barriers like cost, accessibility issues etc*)
6. What difficulties or barriers do you face in delivering care to STDR patients? How do you cope with this?*(Probe: difficulties in delivering care because of inadequate trained  staff, equipment, medicines or because  of heavy crowds so lack the time to spend with patients etc)*
7. What suggestions do you have to improve care delivery for STDR patients?
