# Supplementary material for: ‘I don`t need an eye check-up’. A qualitative study using a behavioural model to understand treatment-seeking behaviour of patients with sight threatening diabetic retinopathy (STDR) in India
Source: PLoS One. 2023 Jun 15;18(6):e0270562. doi: 10.1371/journal.pone.0270562 (PMC10270603; doi:10.1371/journal.pone.0270562)
Supplement: S2 Text — (DOCX) [file pone.0270562.s002.docx]

**SMART India (STDR) study- Code Book**

- Pt-Socio demographics
- HCP-Socio demographics

**CG-Care Seeking**

- CG-Care- Perceptions on reasons for seeking or not seeking care
- CG-Care- Support- no support from other family members
- CG-Care- What can be done to improve care seeking
- CG-Care-Barriers difficulties in following Dr. advice
- CG-Care-CG's role in motivating patient for care seeking
- CG-Care-Concerns about Pt following Dr. advice, financial, poor support, accessibility issues etc
- CG-Care-Nature of care sought

CG-Care- Perceptions on need to seek care in a hospital

- CG-Care-Recent eye test done, where and what was done

**CG-Understanding of illness**

- CG- Understanding-Faith in God fatalistic acceptance
- CG.-Understanding-Awareness about diabetes causing blindness, need for eye check-up
- CG-Understanding- Perceptions on seriousness of eye problem of Pt
- CG-Understanding- Perceptions on what could happen to Pt, its impact in daily life
- CG-Understanding-Frequency of diabetes check done for pt
- CG-Understanding-Information received from HCP about condition
- CG-Understanding-Other diabetes related problems and care sought
- CG-Understanding-Perceptions on nature of eye problem of Pt.

**HCP- Problem Severity**

- HCP-Problem- Awareness of STDR among diabetic patients
- HCP-Problem-Enhancing awareness
- HCP-Problem-Gender related issues
- HCP-Problem-Role of HCPs in improving-creating awareness in people
- HCP-Problem-Seriousness of problem, prevalence, who are at risk

**HCP-Care Provision**

- HCP-Care- Nature of treatment provided
- HCP-Care- Reasons for compliance and non-and compliance to treatment among patients role of social support for compliance
- HCP-Care- Role of family friends in care provision
- HCP-Care-Barriers to treatment
- HCP-Care-Effectiveness of treatment
- HCP-Care-Suggestions for improving care seeking

**Pt- Understanding of problem**

- Pt- Home remedies for diabetes
- Pt.- Understanding- Seriousness of problem, fears of loss of vision
- Pt.-Understanding- Co-morbidities
- Pt.-Understanding- Implication of disease for their daily life
- Pt.--Understanding- Perceptions of what they are suffering from, how do they refer to it
- Pt-Duration of diabetes
- Pt-Not informed about eye problem by opthalmologists
- Pt-Status of vision currently
- Pt-Understanding- Awareness or experiences of any eye related problems

**Pt.- Care Seeking**

- Pt Care-Family support
- Pt Care-Frequency of consultation for diabetes and or eye care, blood test
- Pt. Care- Facilitators that enable care seeking
- Pt.- Care- Nature of advice given by HCPs
- Pt.- Care-Perceptions on need for seeking care
- Pt.-Care- Concerns about following HCP advice
- Pt.-Care-Effectiveness satisfaction with care provided
- Pt.-Care-Nature of care if any sought steps taken to care for self
- Pt-Care- Support from family
- Pt-Care-Barriers and challenges to care seeking

**Pt.-Reasons for people with STDR not seeking care**

- Pt.-Reasons- What can be done to encourage pts to seek care
- Pt-Reason poor susceptibility
- Pt-Reasons financial
- Pt-Reasons- lockdown
